# Synchronous psychological interventions by videoconferencing for caregivers of people with dementia: an integrative review

**DOI:** 10.1590/1980-5764-DN-2021-0069

**Published:** 2022

**Authors:** Maryam Furlan Ayoub, Yara Luana Pereira de Souza, Thiago de Almeida, Deusivania Vieira da Silva Falcão

**Affiliations:** 1 Universidade de São Paulo, Faculdade de Filosofia, Ciências e Letras de Ribeirão Preto, Ribeirão Preto SP, Brazil.; 2 Universidade de São Paulo, Faculdade de Artesm Ciências e Humanidades, São Paulo SP, Brazil.

**Keywords:** Caregivers, Dementia, Internet-Based Intervention, Videoconferencing, Cuidadores, Demência, Intervenção Baseada em Internet, Comunicação por Videoconferência

## Abstract

The COVID-19 pandemic has created the need to develop psychological interventions to support people with dementia and their caregivers in the context of social distancing. This study sought to investigate, systematize, and report results of scientific studies published in the past 5 years on synchronous online psychological interventions using videoconferencing for informal caregivers of people with dementia. The PubMed, BIREME, and Web of Science databases were searched using the descriptors “caregiver,” “dementia,” “online,” and “intervention.” Six international studies were included in the review. Results demonstrated, in general, that this modality of intervention was acceptable, feasible, and promoted benefits for the health, quality of life, and well-being of caregivers. A need was identified for further studies investigating synchronous online interventions that include follow-up and a control group to further the evidence on the effectiveness and feasibility of this type of therapeutic intervention.

## RESUMO.

A pandemia da COVID-19 exigiu o desenvolvimento de intervenções psicológicas para apoiar as pessoas com demência e seus cuidadores no contexto de distanciamento social. No presente estudo, buscou-se investigar, sistematizar e apresentar resultados de pesquisas científicas produzidas nos últimos cinco anos sobre intervenções psicológicas *online* síncronas, por meio de videoconferência, com cuidadores informais de pessoas com demência. Para tanto, utilizaram-se as bases de dados PubMed, Centro Latino-Americano e do Caribe de Informação em Ciências da Saúde (BIREME) e Web of Science, por meio dos descritores “*caregiver*”, “*dementia*”, “*online*” e “*intervention*”. Foram incluídos na revisão seis estudos internacionais que demonstraram, em geral, que essa modalidade de intervenção é aceitável, viável e passível de promover efeitos positivos na saúde, na qualidade de vida e no bem-estar dos cuidadores. Identificou-se a relevância de estudos futuros investigarem intervenções *online* síncronas com a implementação de medidas de *follow-up* e grupo controle, a fim de agregar mais evidências da efetividade e da viabilidade desse tipo de intervenção terapêutica.

## INTRODUCTION

Dementia is a syndrome characterized by decline in one or more cognitive domains, beyond that expected in normal aging. There are an estimated 50 million people living with dementia worldwide and a further 10 million new cases every year, with Alzheimer’s disease (AD) being the most common type^1^. The condition affects not only the individual with the disease but also their caregivers and family members who assist them in carrying out activities of daily living^2^, as well as dealing with the challenges posed by disease progression.

Women, particularly daughters or wives, predominantly perform this role^3^, taking on multiple tasks and sometimes a triple workload that includes formal work, routine housekeeping, and caring for an elderly relative. This situation can lead to difficulties involving communication; division of tasks and relationships among family members; culminating in a high burden associated with stress, anxiety, and depressive symptoms; and disruption in the family balance^4,5^.

Given the numerous deleterious effects on the mental and physical health of caregivers, interventions have been devised aimed at improving the quality of life and well-being of this population. The main intervention approaches include (a) counseling and psychotherapy (individual, group, and family), (b) psychoeducation, (c) multicomponent interventions (combining several intervention strategies to address a range of caregiver needs), and (d) interventions based on full attention (employing mindfulness strategies and other meditation techniques)^6^.

Interventions for caregivers can be in-person or face-to-face, by telephone, or over the Internet^7^. Those conducted on the Internet can be synchronous and/or asynchronous. Synchronous interventions offer the advantage of having a prescheduled time and day, helping the caregiver prepare for and take part in interventions. Asynchronous interventions have the advantage that they can be performed at any time of the day, allowing the caregiver to engage in the activities when free to do so. However, this latter approach requires self-discipline and good organizing skills in order to be effective. Huis in het Veld et al.^8^ delivered an asynchronous intervention via email and videos involving 81 families of caregivers of persons with dementia. The study failed to obtain favorable results using this approach, reporting no improvement in the quality of life of participants. This poor outcome may have been due to the low engagement of family members regarding the materials delivered.

Boots et al.^9^ conducted a systematic review of online asynchronous and synchronous interventions for this population, concluding that Internet-based interventions are promising and can help improve caregiver well-being. The authors, however, highlighted that further studies are needed to develop and support protocols, as well as to confirm these positive findings.

The COVID-19 (Coronavirus disease-19) pandemic, caused by the novel coronavirus (SARS-CoV-2), has created a need to conduct health consultations remotely so as to reduce the risk of cross-contamination through close personal contact^10^. This situation has prompted the need to adapt interventions for remote application and apply them online by videoconferencing (VC). Referred collectively as “telehealth”, these video calls take place live using software^11^ through which health professionals provide patients with advice and address their health needs.

Against this backdrop, and given the current importance of implementing online synchronous interventions for caregivers of persons with dementia, the objective of this study was to investigate, systematize, and present the results of studies published in the past 5 years on group and/or individual therapeutic interventions by VC for informal caregivers of this patient group.

## METHODS

The present integrative review of the literature entailed a detailed search of the PubMed, BIREME, and Web of Science databases between September and October 2020. Relevant articles published in the past 5 years were searched. The search string employed the following English descriptors combined using Boolean operator AND: *caregiver, dementia*, *online*, and *intervention*. Articles were first screened based on title, with subsequent analysis of their respective abstracts.

After reading of abstracts, articles that met the following criteria were included: (1) original article, (2) published in Portuguese or English, (3) published in the past 5 years (January 2015 to September 2020) in indexed journals held on the selected databases, (4) studies involving the application of therapeutic group or individual interventions in human subjects online by VC, and (5) studies with interventions whose target population was informal caregivers of persons diagnosed with dementia. Criteria for study exclusion were (1) studies not including VC sessions with informal caregivers of persons with dementia, (2) studies not yet concluded, and (3) review articles, monographs, dissertations, theses, book chapters, or descriptions of intervention protocols.

**Figure 1:**
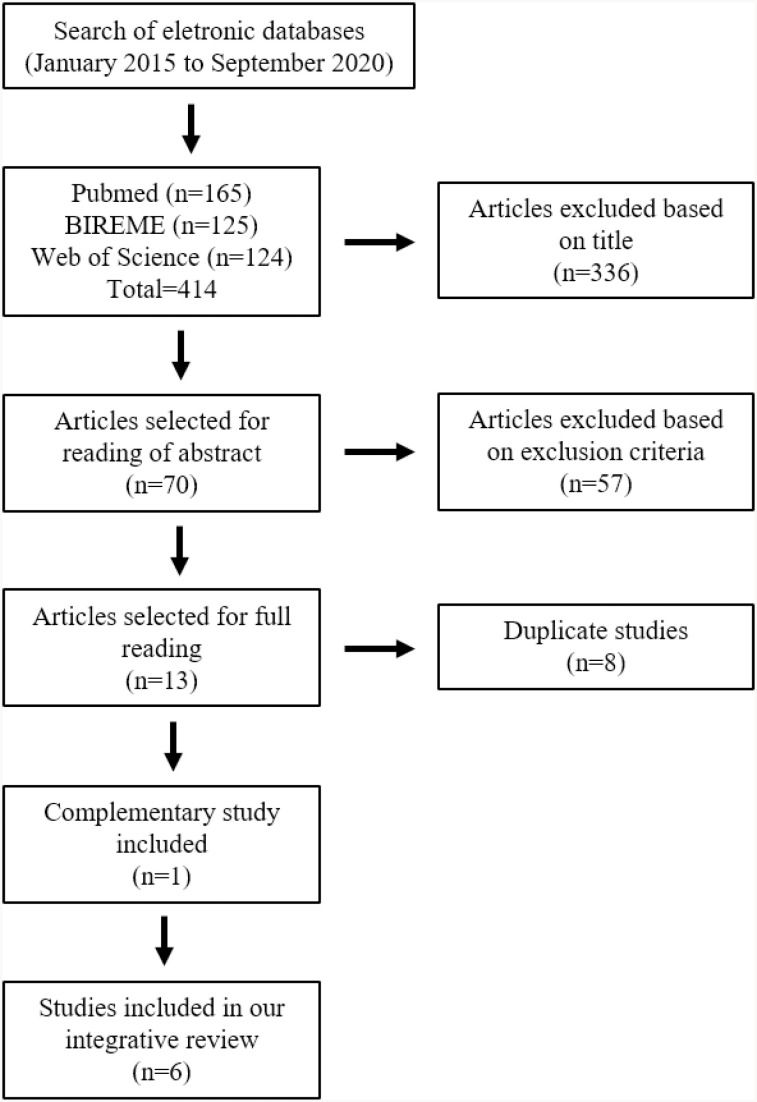
Flow diagram of literature search.

The initial search of the databases led to the retrieval of 414 scientific studies: PubMed (n=165), BIREME (n=125), and Web of Science (n=124). After examination of titles, 70 studies were selected for reading of their respective abstracts. Based on the inclusion criteria, 13 articles were then selected for reading in full. After rejection of articles duplicated among the databases (n=8), five scientific studies were selected for inclusion in the review. A further complementary study was identified from references, giving a total of six articles for inclusion in the present review (Figure 1).

Two independent evaluators (first authors of the study) carried out a thorough analysis of the articles selected for full reading in order to appraise the suitability for inclusion in this integrative review of the literature, according to the abovementioned predefined selection criteria. The results of the two analyses by the evaluators were fully concordant on the studies shortlisted for inclusion in the review. The article contents were extracted according to the following categories: (1) author/year, (2) country of origin, (3) methodological design employed (staggered randomized waitlist, single group or with control group, involving both pre- and posttests or posttest only, including focus group or otherwise), (4) participants who underwent intervention, (5) description of intervention, (6) main findings, and (7) conclusion.

## RESULTS

Of the six articles included in the review^12-17^, most were conducted in the United States^12,14-17^, while one study was carried out in Australia^13^. With the exception of the study by Moskowitz et al.^16^, in which interventions were delivered individually, all studies^12-15,17^ involved group-based interventions.

The majority of interventions were psychoeducational^14-17^, while one investigation involved an emotion-support intervention^13^ and another focused on educational and psychosocial support^12^. Despite the variability in the types of intervention delivered, all sessions using VC (group or individual) were conducted by an instructor or facilitator for all studies (Table 1).

**Table 1:** Online synchronous interventions for informal caregivers of people with dementia.

Author(s)	Country	Design	Participants	Intervention	Main findings	Conclusions
Austrom et al.^12^	The United States	Pilot study involving single group and assessments at pre- and posttests, and posttest focus group.	Family caregivers of persons with dementia (n=5).	Psychosocial educational web-based VC support group held once a week for 6 months. The group was facilitated by first author of the study and had general format comprising presentations, followed by educational instructions, questions and answers, and sharing and support. Three guest speakers also covered content in genetics, genetic counseling, elder law issues, and community-based social services. Alzheimer’s Association brochures and educational modules were also made available.	Improvement in several aspects of mental health were noted in participants at posttest relative to pretest, with the following mean differences in scores: anxiety (1.5 points), depression (3.3 points), physical health (-2.7 points), and controlling upsetting thoughts and response to disruptive behavior (−1.5 and −9.5 points, respectively). Qualitative data in form of feedback from program participants showed generally very positive views (e.g., appreciation for convenience of remote format, guest speakers, and technical support of research team).	Web-based support groups for caregivers using online tools proved feasible, acceptable, and low-cost, where pros outweighed cons (e.g., missing being in person with other caregivers) of this format.
Banbury et al.^13^	Australia	Staggered randomized waitlist design.	Primary dementia caregivers socially or geographically isolated (n=69).	Support intervention by group VC for 6 weeks. There were 16 groups and sessions lasted an average of 90 min each. Sessions were led by a facilitator who facilitated discussions on different topics, and participants were able to share their experiences and knowledge. The program also provided IT support via a technician.	Most participants had no prior experience of using VC. However, following completion of the program, they reported positive views about the tool. Although all adjusted to communicating digitally, some preferred in-person meetings. Other participants, however, reported feeling more comfortable meeting by VC. Most (n=51) indicated they would like to continue meeting on a self-organized basis, and eight groups self-organized to meet.	Remote support groups based on telecommunication technology are telehealth interventions that may have the potential to develop networks among isolated caregivers of people with dementia.
Griffiths et al.^14^	The United States	Pilot study involving single group with pre- and posttests.	Family caregivers of persons with dementia (n=57).	“Tele-Savvy” (TS) comprised six group sessions with four to eight caregivers by VC, with weekly 75 min sessions. Each VC session was dedicated to a key topic in a discussion and lecture style. Modules were delivered of six asynchronous daily 5–15 min long videos per week. Participants were provided with a manual and a workbook that complemented and reinforced their learning.	Apparently, TS contributed to significant improvement in caregivers´ well-being for burden (p<0.01), depressive symptoms (p<0.05), and self-reported competence (p<0.001). There was also a significant decrease in average BPSD frequency (p<0.05) and number of BPSD that occurred more often p<0.01).	The TS is a feasible, efficacious psychoeducation program for promoting improvement in mental health of caregivers and reducing BPSD of care recipients with dementia.
Kovaleva et al.^15^	The United States	Intervention involving single group and posttest assessment.	Unpaid family caregivers of persons living with dementia (n=36).	TS comprised seven group sessions with weekly 60–80 min sessions. Instructors led lectures and discussions and sharing of caregiver experiences. Prerecorded video classes were made available daily, presenting educational content. A caregiver manual was also provided to promote self-efficacy.	The intervention proved viable and acceptable by participants, most of whom preferred the online mode. Almost all participants reported connectedness with other caregivers and instructors. Many participants requested inclusion of specific content (e.g., information on each dementia stage) and greater content diversity in videos (e.g., inclusion of actors of other ethnicities).	The TS addresses the needs of caregivers, promotes connectedness in in-person interactions, with benefit of convenient delivery. However, the program should by split into modules according to the different profiles of caregivers (e.g., dementia stage based).
Moskowitz et al.^16^	The United States	Randomized clinical trial with control group and pre- and posttests.	Primary caregivers of persons with degenerative dementia (n=170). Intervention group (n=86) Control group (n=84)	“Positive emotion regulation intervention” (LEAF, Life Enhancing Activities for Family caregivers) comprising six synchronous online sessions individually delivered by trained facilitators. During the course of sessions, emotion regulation skills were taught to increase positive emotion levels.	Intervention group showed significantly greater improvements in outcomes of dependent variables at posttest compared to control group: positive emotion (d=0.58; p<0.01), depression (d=-0.25; p=0.02), anxiety (d=-0.33; p<0.01), physical (d=0.24; p=0.02), and positive aspects of caring (d=0.36; p<0.01). According to analyses of multilevel moderate mediation, increased positive emotion significantly mediated the effect of the intervention on depression.	The intervention proved feasible and acceptable and promises to be effective for family caregivers of persons with dementia experiencing stress associated with caregiving.
Paun and Cothran^17^	The United States	Pilot study involving single group with pre- and posttests.	Family caregivers of individuals with AD and related dementias residents of long-term care institution (n=5).	Online group-based intervention “Chronic Grief Management – A Live-Streaming, Online Intervention” (CGMI-V) comprising eight sessions by VC, with one session per week averaging 60 min each. The group addressed three central topics: “knowledge, communication abilities/conflict resolution and grief management.”^17^	In general, participants were able to use technology without major problems, positively assessing small groups, and encountered no problems connecting emotionally with one another. Participants also reported the group made them feel less alone and guilty for institutionalizing the elderly relative.	CGMI-V is a feasible intervention for delivery online and led by a professional. Also, the intervention has the potential to promote a significant effect on chronic grief and other aspects of mental health of caregivers of persons with dementia.

BPSD:Behavioral and Psychological Symptoms of Dementia; VC: videoconferencing.

Overall, studies showed that interventions by VC in informal caregivers of persons with dementia were feasible, and all programs implemented support measures to assist participants with the use of the technology. These measures included making available devices to access sessions by VC, such as tablets and personal computers^12,13,16,17^, individual training/guidance^12-17^, home-based visits to set up the computer software needed to run VC sessions or rectify Internet or hardware issues^12^, and provision of written instructions^14,15,17^.

Participant retention rates during intervention were high for all studies. More specifically, the study by Moskowitz et al.^16^ (total of participants=170) had a high participation retention rate throughout the interventions for both the experimental group (89.0%) and control group (92.8%). Similarly, only three participants dropped out of the study by Banbury et al.^13^ (total of participants=69), six in the study of Kovaleva et al.^15^ (total of participants=36), seven in Griffiths et al.^14^ (total of participants=64), and only one participant dropped out of the study by Austrom et al.^12^ (total of participants=5). Even better results were reported by Paun and Cothran^17^,with none of the five participants discontinuing.

All the studies showed acceptability of the interventions, as evidenced by quantitative and qualitative results postintervention (Table 1). In this regard, Griffiths et al.^14^ found significant improvement in scores on several measures of mental health of participants at posttest versus pretest (e.g., caregiver burden, depressive symptoms, and self-reported competence). In addition, positive effects were also evident at posttest in care recipients, such as a significant decrease in number of behavioral and psychological symptoms of dementia (BPSD) that occurred daily or more often and average BPSD frequency. Moreover, Austrom et al.^12^ also observed posttest improvement on measures of caregiver well-being (e.g., anxiety, depression, physical health, controlling upsetting thoughts, and response to disruptive behavior) (Table 1).

Moskowitz et al.^16^ noted a significantly greater improvement in scores for positive emotion, depression, anxiety, physical health, and positive aspects of care among participants delivered the intervention individually for regulating emotion (intervention group) versus control subjects (waitlist). The study by Banbury et al.^13^, involving an intervention with socially or geographically isolated informal dementia caregivers, showed that, despite having little prior experience of using VC technology, most participants rated the tool positively. In addition, after completing the intervention, eight groups of carers continued to meet on a self-organized basis (Table 1).

With regard to the online modality of interventions, qualitative data from the study by Paun and Cothran^17^ revealed that the participants experienced no problems emotionally engaging with other participants and felt supported and less guilty about their decision to institutionalize family members with dementia. Mirroring these findings, most of the participants of the study by Kovaleva et al.^15^ reported connectedness with the other carers and instructors and preferred the online mode of the program. Furthermore, the feedback given by participants on the intervention in the study of Austrom et al.^12^ were also generally positive, with caregivers appreciative for convenience of being able to take part in the support group remotely (Table 1).

## DISCUSSION

The objective of this review was to identify and present the results of studies published in the past 5 years (from January 2015 to September 2020) on synchronous online interventions by VC involving informal caregiver of people with dementia. This mode of delivery was found to be a valid approach for promoting access and adherence to psychoeducational and emotional support programs, particularly among caregivers unable to attend sessions in person. In addition, the VC interventions proved both feasible and acceptable, with potential to have positive effects on the quality of life of caregivers.

Of the six articles included in the review, only one^16^ involved delivery of individual interventions in informal dementia caregivers. The study in question^16^ entailed implementation of a positive emotion regulation program and promoted significantly greater improvement in caregivers’ mental health than that of control subjects (waitlist). However, the authors pointed out that, although delivery individually was likely a factor contributing to the high participant retention rates observed, the strategy is relatively high cost and difficult to reliably replicate on a large scale^16^. Thus, while individual remote delivery can aid access to participants for intervention implementation and data collection by researchers, group interventions appear to be more feasible than individual interventions.

While the studies reviewed were heterogeneous for some aspects of the interventions (e.g., number of participants, number of sessions, and aims), they were similar in other respects (e.g., target population, use of VC, sessions delivered by instructors/facilitators, and assistance on using the technology). Also, for all studies involving group interventions, these groups were closed, that is, further participants were not included over and above the initial group recruited. This homogeneity across the studies aided the analysis of their respective results.

Overall, the studies had high rates of retention of participants engaged in the intervention programs. This satisfactory adherence may have been due, in part, to the assistance given to participants on how to access and use the technology adopted, including provision of electronic devices, written instructions, and individual training. In the scientific literature, most previous studies in this field have involved face-to-face interventions, which are indeed the most typical modality employed. Therefore, the help given to participants in using the technology (e.g., VC software) appears to be a key element for success, as highlighted by half of the studies reviewed^12,13,17^, where caregivers positively rated the help provided on using the technology.

The studies reported positive effects on carers who underwent the interventions. The studies in which analyses of quantitative data were carried out^12,14,16^ confirmed improvements in the quality of life of caregivers, particularly with respect to depressive symptoms, anxiety, and physical health. Qualitative data also revealed the positive effects of interventions: reduced feelings of loneliness and blame^17^, connectedness among participants^15,17^, and the establishing of interpersonal networks among isolated caregivers after conclusion of the group intervention.^13^ Taken together, these results reiterate the potential of online interventions using VC for promoting health gains (physical and mental) and forming connections among participants of group programs.

Similarly, a previous review^18^ also showed that online interventions for caregivers of people with dementia promoted positive effects among caregivers, showing potential to improve well-being. The study also demonstrated that both the direct contact among participants and presence of a professional facilitating the intervention appeared to be key factors favoring the mental health of participants and attainment of the goals of the interventions, respectively. In this respect, all the studies included in this review^12-17^ involved at least one of these factors, whereas most^12-15,17^(5/6 articles) involved both. The presence of these factors is believed to have played a role in the positive results found for all the interventions reviewed.

Notably, only one study^16^ included a control group for the purposes of comparison against the intervention group. The lack of a control group for the remaining studies in which quantitative analyses were carried out compromised the robustness of the method, precluding the drawing of causal inferences between the independent (intervention) and dependent (e.g., anxiety, depression, and burden) variables assessed. Moreover, none of the studies included or reported results on the follow-up of participants, rendering it impossible to determine whether the postintervention gains persisted over time. A follow-up analysis would have allowed assessment of the potential of interventions to promote long-term effects on caregivers.

Kovaleva et al.^15^ found that the heterogeneity of the care situations among participants was identified by some individuals as a factor hampering a stronger connection with the other participants, while many carers expressed the desire for more information on the specific stages of the disease. The authors concluded that the program groups should be divided according to the profile of participants, for example, by disease stage of the care recipient. This finding suggests future online group psychoeducational interventions should be designed that taken these aspects into account, thereby promoting greater group cohesion and stronger connectedness among participants and allowing the specific needs of each stage to be met.

Only one of the studies reviewed^12^ reported availability of electronic devices and Internet access to all participants. Hence, although online remote synchronous interventions allow participation of a number of caregivers unable to attend sessions in person (e.g., during pandemics or because patient cannot be left alone), this raises questions on the more socioeconomically vulnerable caregivers who do not enjoy access to the Internet or electronic devices required for interventions. Future studies should explore feasible effective ways of including these individuals in their intervention programs for dementia caregivers.

Although the studies reviewed confirmed the feasibility and acceptability of online synchronous interventions by VC for caregivers of people with dementia, this review involved a relatively small number of studies (n=6), reducing the potential for generalization of results. Future studies could combine different key words to search a broader set of scientific databases and yield additional studies. Another study limitation was, of those studies incorporating quantitative data measure (n=3), only one employed a control group, reducing the power of results to some extent.

Investigations could be conducted that include follow-up of participants to determine whether the benefits promoted by interventions are maintained over time. Lastly, online synchronous interventions using VC for dementia caregivers proved feasible, exhibiting potential to enhance the quality of life and well-being of this population. Future studies should be conducted that report follow-up results and systematically investigate interventions of this modality with the inclusion of a control group.
